# ﻿Integrative delimitation of a new *Epeorus* (*Caucasiron*) (Ephemeroptera, Heptageniidae) from the Caucasus with a supplement to the identification guide of Caucasian and Irano-Anatolian species

**DOI:** 10.3897/zookeys.1214.131266

**Published:** 2024-10-09

**Authors:** Ľuboš Hrivniak, Pavel Sroka, Gencer Türkmen, Alexander V. Martynov, Jindřiška Bojková

**Affiliations:** 1 Department of Botany and Zoology, Faculty of Science, Masaryk University, Kotlářská 2, 61137 Brno, Czech Republic Masaryk University Brno Czech Republic; 2 Biology Centre of the Czech Academy of Sciences, Institute of Entomology, Branišovská 31, 370 05 České Budějovice, Czech Republic Biology Centre of the Czech Academy of Sciences, Institute of Entomology České Budějovice Czech Republic; 3 Department of Biology, Faculty of Science, Hacettepe University, 06800 Beytepe, Ankara, Turkiye Hacettepe University Ankara Turkiye; 4 National Museum of Natural History, National Academy of Sciences of Ukraine, Bohdan Khmelnytsky str., 15, 01030, Kyiv, Ukraine National Museum of Natural History, National Academy of Sciences of Ukraine Kyiv Ukraine

**Keywords:** Aquatic insects, species delimitation, taxonomy

## Abstract

As part of our detailed study of the Caucasian mayfly fauna, we describe Epeorus (Caucasiron) abditus**sp. nov.**, a new species of the genus EpeorusEaton, 1881,subgenusCaucasiron Kluge, 1997, based on larvae collected in Türkiye, Georgia, and Russia. We use several methodological approaches to delimit the new species by analysing COI sequence data and larval morphology. We provide a comparison with related taxa and diagnostic characters allowing determination of the larvae. We also update the identification key for the Caucasian species of E. (Caucasiron) with E. (C.) abditus**sp. nov.** and two recently described species, E. (C.) hyrcanicus Hrivniak & Sroka, 2021 and E. (C.) tripertitus Hrivniak & Sroka, 2022.

## ﻿Introduction

The biota of the Caucasus biodiversity hotspot is extraordinarily diverse (Mittermeier et al. 2011) and mayflies (Ephemeroptera) are no exception. Currently, 130 species from 15 families and 33 genera are known from the Caucasus, almost half of which (61 species) are considered endemic (Hrivniak 2020). The genus *Epeorus* Eaton, 1881 is one of the most diversified mayfly genera in the Caucasus region. The larvae inhabit cold and well-oxygenated streams and rivers with stony substrate and are relatively common in the region (Bojková et al. 2018; Hrivniak et al. 2018). Considering the large body size of mature larvae, which can exceed 20 mm in some species, such as E. (Caucasiron) magnus (Braasch, 1978), they represent a charismatic and conspicuous group of mountain aquatic biota. Except for a single species, Epeorus (Epeorus) zaitzevi Tshernova, 1981, all Caucasian Epeorus species belong to the subgenus Caucasiron Kluge, 1997 (Hrivniak et al. 2021), which encompasses 17 species known from the Caucasus and the neighbouring mountains (Pontic, Taurus, and Zagros Mountains) (Hrivniak et al. 2022).

The geographic distribution of E. (Caucasiron) is split into two areas, the Caucasus and Central-East Asia, which includes the Tian Shan, the Himalayas, and mountain ranges in the Yunnan-Guizhou Plateau in south-west China (Braasch 2006; Chen et al. 2010; Kluge 2015; Ma et al. 2022). The latter appears to be less diversified and species-rich, although it has been studied less intensively.

Current phylogenetic analyses suggest that species richness may be even higher in the Caucasus, as cryptic diversity was detected within E. (C.) znojkoi Tshernova, 1938 and E. (C.) tripertitus Hrivniak & Sroka, 2022 (Hrivniak et al. 2020a, 2022), and some unexplored lineages were found. In this study, we investigate the identity of several specimens that were rarely found in Türkiye, Georgia, and Russia during our extensive sampling in the area in 2013–2019. To test whether they represent a new species, we use various molecular species delimitation tools and comparative morphology. Additionally, we extend an identification guide for E. (Caucasiron) larvae published by Hrivniak et al. (2020b) to include the species described after 2020 and allow correct identification of all E. (Caucasiron) species from the Caucasus and adjacent Mediterranean and Irano-Anatolian regions.

## ﻿Material and methods

The material used for this study was collected in Russia (2013), Türkiye (2016), and Georgia (2016, 2019) using hydrobiological hand net. All specimens were preserved in 75–96% EtOH and are deposited in the collections of the Biology Centre of the Czech Academy of Sciences, Institute of Entomology, České Budějovice, Czech Republic (IECA). The material of other E. (Caucasiron) species used for morphological comparisons was obtained from the IECA collection.

### ﻿Morphological examination

Parts of specimens were mounted on microscopic slides using HydroMatrix (MicroTech Lab, Graz, Austria) mounting medium. To remove the muscle tissue for an investigation of the cuticular structures, specimens were left overnight in a 10% solution of NaOH prior to slide mounting. Drawings were made using a stereomicroscope Olympus SZX7 and a microscope Olympus BX41, both equipped with a drawing tube. Photographs were obtained using Leica DFC450 camera fitted with macroscope Leica Z16 APO and stacked in Helicon Focus ver. 5.3 X64. All photographs were subsequently enhanced with Adobe Photoshop ver. CS5. Morphological diagnostic characters for the description of a new species were adopted from Hrivniak et al. (2020b).

### ﻿DNA extraction, PCR, sequencing and alignment

Total genomic DNA of four specimens (labelled as A1–A3 and A6) was extracted from legs using the DEP-25 DNA Extraction Kit (TopBio) and DNeasy Blood & Tissue Kit (Qiagen), both according to the manufacturer’s protocol. Mitochondrial cytochrome oxidase subunit I (COI) was sequenced according to Hrivniak et al. (2017). COI sequences of other E. (Caucasiron) species used for molecular comparisons were obtained from Hrivniak et al. (2017, 2019, 2020c, 2021, 2022). The PCR amplification of COI and reaction volumes was carried out as described in Hrivniak et al. (2017). Sequences were assembled in Mega X (Kumar et al. 2018) and aligned in Jalview (Waterhouse et al. 2009) using the Mafft algorithm. Newly obtained sequences were deposited in GenBank with accession numbers (GB) PP987168–PP987171.

### ﻿Molecular species delimitation

Molecular delimitation of species was performed using the single threshold General Mixed Yule Coalescent model (GMYC, Pons et al. 2006; Fujisawa and Barraclough 2013), Multi-rate Poisson tree processes for single-locus (mPTP; Kapli et al. 2016) and the Assemble Species by Automatic Partitioning (ASAP; Puillandre et al. 2021). GMYC, mPTP, and ASAP were performed by the online servers https://species.h-its.org/gmyc/, https://bio.tools/mptp and https://bioinfo.mnhn.fr/abi/public/asap/, respectively. The COI gene tree for GMYC and mPTP was reconstructed using BEAST ver. 2 (Bouckaert et al. 2014) with settings described in Hrivniak et al. (2020c). The dataset included all described species from the subgenus Caucasiron distributed in the Caucasus and adjacent regions. Two analyses were running on CIPRES Science Gateway (Miller et al. 2010) for 200 million generations sampled every 20 000 generations. Convergence and effective sample size (ESS > 200) were verified using Tracer ver. 1.7 (Rambaut et al. 2018). The first 10% of trees from each run were discarded as burn-in. Files from both independent runs were combined using LogCombiner ver. 2.6.7. The maximum clade credibility tree was constructed using TreeAnnotator ver. 1.8.4 with default settings. The input dataset for ASAP comprised sequences aligned in a fasta file. The simple pairwise genetic distances were selected, and other settings were default. Inter- and intraspecific pairwise genetic distances were calculated in MEGA X.

## ﻿Results and discussion

### ﻿Molecular species delimitation

The final COI alignment contained 97 sequences, 631 base pairs and 197 parsimony informative positions. The single threshold GMYC model estimated 20 species (CI = 12–28) consisting of 19 ML clusters and one singleton. Epeorus (Caucasiron) abditus sp. nov. was delimited as a distinct species. The mPTP method and the distance-based ASAP analysis also delimited E. (Caucasiron) abditus sp. nov. as a distinct species (Fig. 2). The monophyly of all species clusters was supported (PP = 1).

Pairwise genetic distances between E. (Caucasiron) abditus sp. nov. and other E. (Caucasiron) species ranged from 8.33% in E. (C.) magnus to 15.93% in E. (C.) shargi Hrivniak & Sroka, 2020. Genetic distances within E. (Caucasiron) abditus sp. nov. varied between 0.33 and 1.64%.

### ﻿Taxonomy

Epeorus (Caucasiron) abditus sp. nov. is attributed to the subgenus Caucasiron within the genus *Epeorus* based on the presence of projection on the costal rib of gill plates II–VII (Fig. 5G, arrow), and the presence of medio-dorsally directed hair-like setae located on the anterior margin of the head (see Kluge 2015 for a revision of the subgenus).

#### ﻿ Epeorus (Caucasiron) abditus

Taxon classificationAnimaliaEphemeropteraHeptageniidae

Hrivniak & Sroka sp. nov.

C52E90E5-6939-5E37-A555-1C76AB307B2C

https://zoobank.org/AF0C3D08-A97C-483C-A766-9B3F9CFE6113

Figs 4 , 5

##### Type material.

***Holotype*** • female larva (GB: PP987170), Türkiye: Artvin Province, Camili Village, unnamed mountain stream, 1599 m a.s.l.; 41°24'04"N, 42°24'04"E; code: CAM 6, 25.7.2016, G. Türkmen leg.

***Paratypes*** • 1 larva (mounted on slide), same data as holotype • 1 larva (GB: PP987171; mounted on slide), Georgia: Adjara, Kobuleti district, vicinity of Khino (Didvake) village, Kintrishi River, 792 m a.s.l.; 41°43'01"N, 42°02'41"E; code: No6, 19.4.2013, A.V. Martynov leg • 1 larva (GB: PP987168, mouthparts mounted on slide), Georgia: Kakheti Province, South of Alazani Pass, Stori River, 1514 m a.s.l.; 42°14'35.1"N, 45°29'44.5"E; code GEO60/2019, 3.5.2019; Ľ. Hrivniak leg • 1 larva, Russia: Kabardino-Balkaria, vicinity of Terskol village, left tributary of Baksan River, 2192 m a.s.l.; 43°14'31"N, 42°33'49"E; 19.5.2013, V.V. Martynov leg • 2 larvae (one barcoded, GB: PP987169), Russia: Kabardino-Balkaria, vicinity of Tyrnyauz village, right tributary of Baksan River, 1904 m a.s.l., 43°21'N, 42°52'E; 19.5.2013, V.V. Martynov leg.

Type material is deposited in IECA.

##### Etymology.

The species name *abditus* (Latin) means hidden. It refers to rare distribution and morphological similarity with related species.

##### Distribution and habitat preferences of larvae.

**Figure 1.**
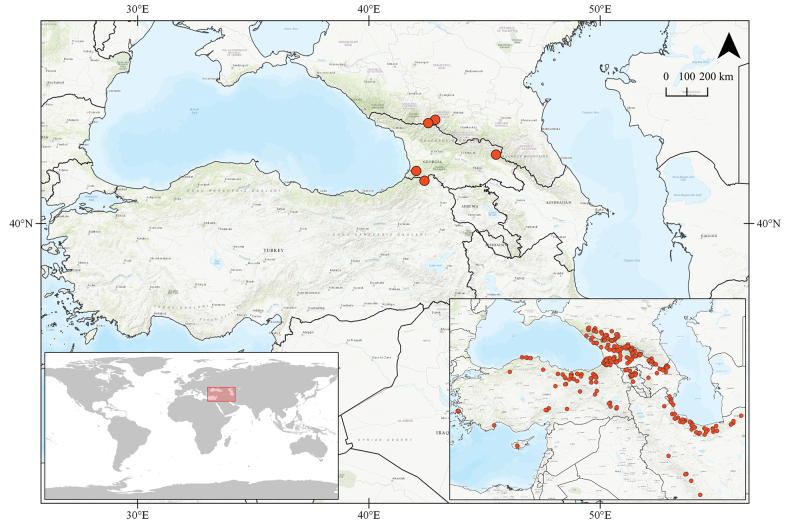
Distribution of Epeorus (Caucasiron) abditus sp. nov.; global localisation of a study area (lower left corner) and our sampling sites investigated between 2008–2019 (lower right corner).

Epeorus (Caucasiron) abditus sp. nov. has relatively wide distribution in the Caucasus region but appears to be relatively rare due to low number of specimens obtained by extensive sampling. They were found in the Pontic Mountains and the Lesser Caucasus (northeast Türkiye and southwest Georgia), and the central (Russia: Kabardino-Balkaria) and eastern (Georgia: Kakheti) parts of the Greater Caucasus (Fig. 1). The larvae were found in low abundance in cold and clear streams and rivers between 792 and 2192 m a.s.l. on stony bed substrate in turbulent flow (Fig. 3). They were not recorded in urban and agricultural areas within the region, where many localities were investigated. Larvae co-occurred with E. (C.) znojkoi, E. (C.) alpestris (Braasch, 1979), E. (C.) magnus.

**Figure 2.**
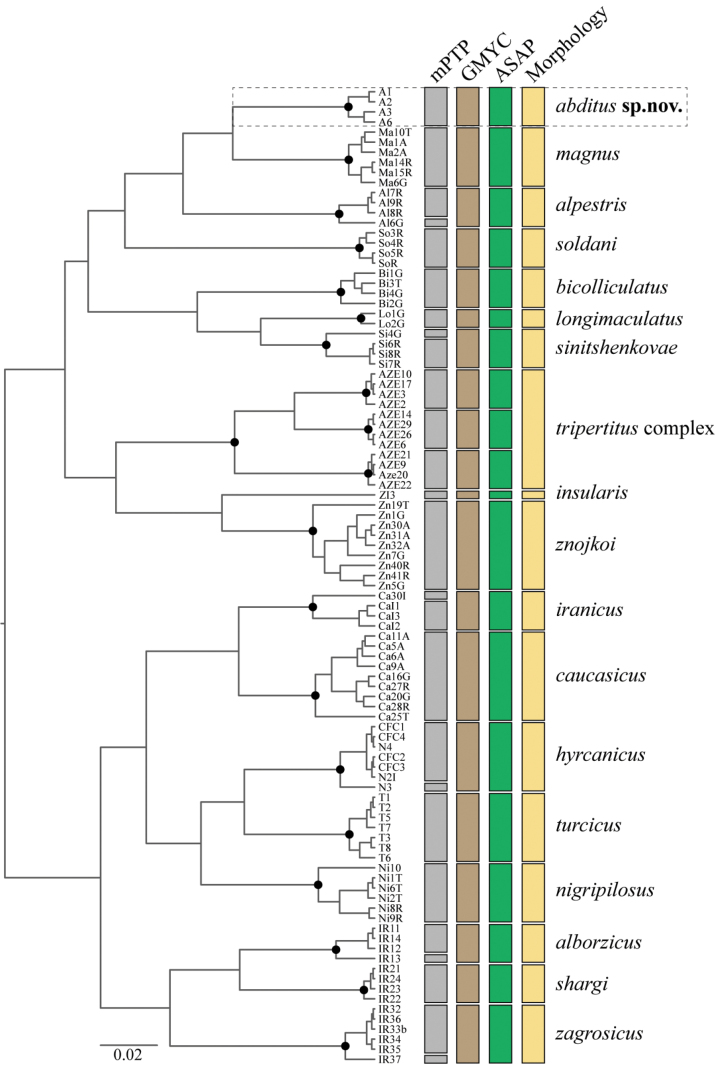
COI gene tree with results of molecular species delimitation tools and larval morphology according to Hrivniak et al. (2020b). Black points correspond to posterior probability 1. Delimitation of Epeorus (Caucasiron) abditus sp. nov. is highlighted by the dashed frame.

**Figure 3.**
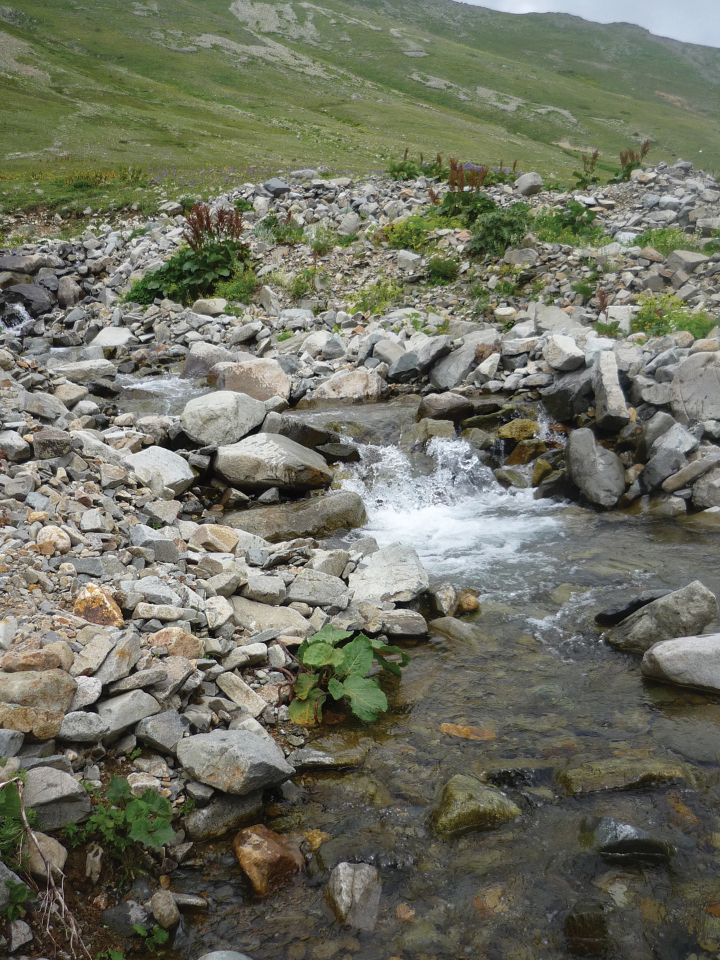
The habitat (type locality) of Epeorus (Caucasiron) abditus sp. nov. from northeast Türkiye (photo: G. Türkmen).

##### Description of larva.

General colouration of larvae yellowish brown with dark brown maculation. Body length of late instar larvae: ca 13.3 mm (female), 11.1–11.25 mm (male). Length of cerci approximately 1.2× body length.

**Figure 4.**
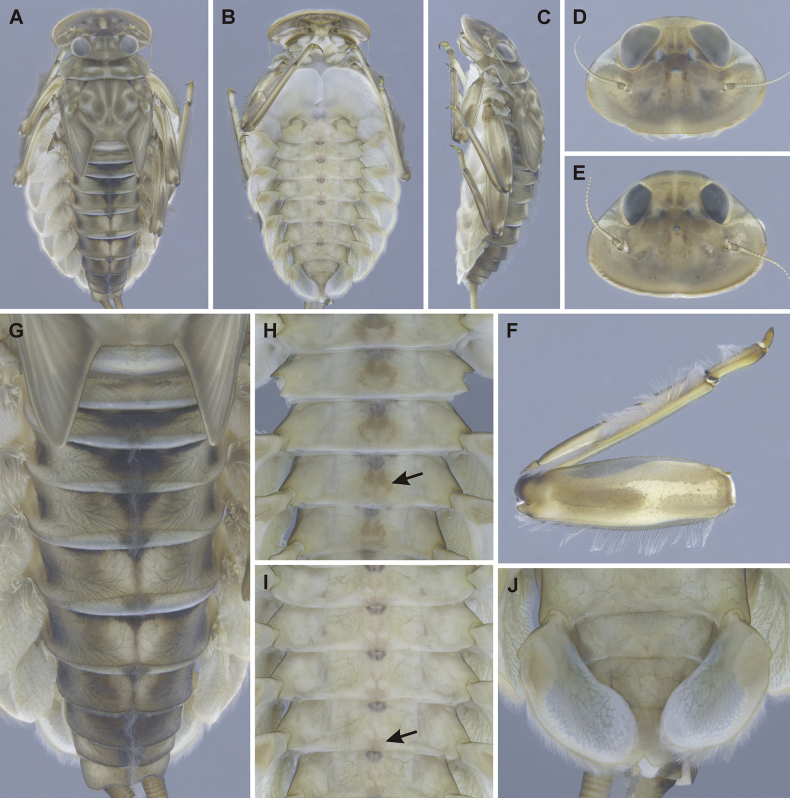
Epeorus (Caucasiron) abditus sp. nov., larva **A** habitus in dorsal view **B** habitus in ventral view **C** habitus in lateral view **D** head of male in dorsal view **E** head of female in dorsal view **F** middle leg in dorsal view **G** abdominal terga **H, I** abdominal sterna II–VI (arrow points on median maculation) **J** gills VII (in natural position from ventral view).

***Head*.** Shape oval to trapezoidal. Anterior and lateral margin rounded, posterior margin rounded in female (Fig. 4E) and slightly rounded in male (Fig. 4D). Head dimensions of late instar larvae: length ca 4.5 mm, width ca 3.2 mm in female; length ca 4.05 mm, width 2.75–2.8 mm in male. Head width/length ratio: 1.46–1.48 in female; 1.46–1.51 in male.

Colouration of head: dorsal surface with pair of elongated maculae located along epicranial suture; pale stripes extending from lateral ocelli to lateral edges of head; blurred (or rectangular) macula between ocelli; rounded maculae anterolateral of lateral ocelli; blurred (or triangular) maculae near inner edges of compound eyes; pair of stripes (or scattered smaller maculae) located anteriorly from median ocellus (Fig. 4D, E). Compound eyes grey to brownish to black. Ocelli blackish. Antennae yellowish brown, scapus and pedicellus darkened. Hair-like setae along anterior margin of head extend to lateral margins. Dorsal surface of head covered with fine hair-like setae and sparsely distributed stick-like setae. Sparse longer and fine hair-like setae located posteriorly to eyes.

**Figure 5.**
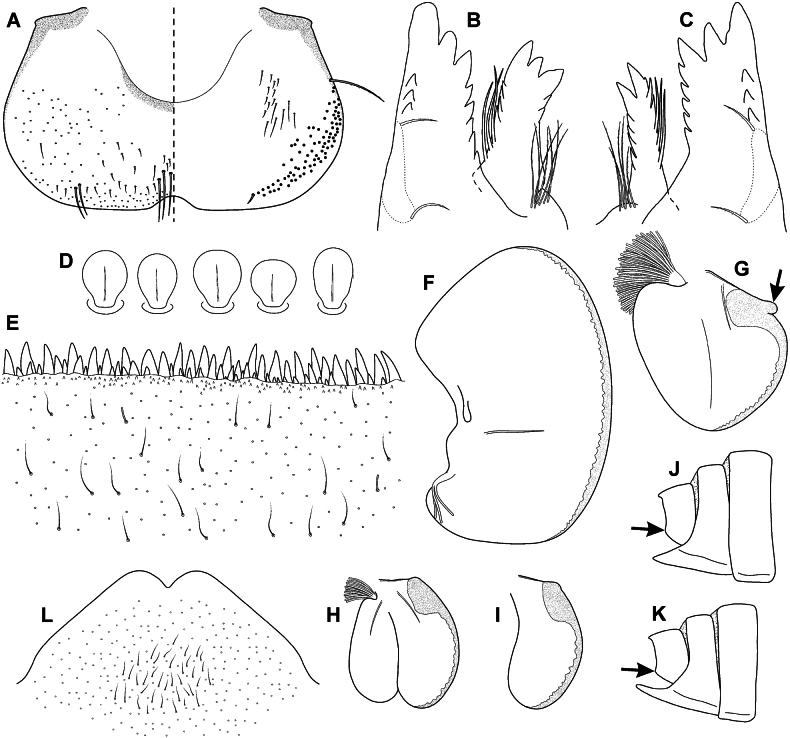
Epeorus (Caucasiron) abditus sp. nov., larva **A** labrum (left half in dorsal view, right half in ventral view; black dots refer to range of setae) **B** incisors of left mandible **C** incisors of right mandible (dashed polygons on outer edge of both mandibles refer to range of setae) **D** setae on dorsal surface of femora **E** surface and posterior margin of abdominal tergum VII **F** gill I **G** gill III (arrow points to projection on costal margin) **H** gill VII (flattened on slide) **I** gill VII (in natural position from ventral view) **J, K** abdominal segments VIII–X in lateral view (arrow points on postero-lateral projection) **L** sternum IX of female.

***Mouthparts*.** Labrum (Fig. 5A) widened anteriorly, with anterior margin slightly rounded or nearly straight (in dorsal view), lateral angles rounded. Dorsal surface (Fig. 5A, left) sparsely covered with hair-like setae and short bristle-like setae; 5–6 longer bristle-like setae located antero-medially and two antero-laterally. Epipharynx with longer, shortly plumose bristles situated along lateral to anterior margin (Fig. 5A, right; range of setation figured as large black dots), and brush of fine hair-like setae medially (not figured); ventral surface with group of 10–16 setae of various size located medio-posteriorly. Outer incisors of both mandibles with three apical teeth (Fig. 5B, C). Inner incisor of left mandible with three apical teeth, right inner incisor bifurcated. Outer edge of both mandibular incisors with numerous setae (range of setae marked with dashed polygons).

***Thorax*.** Pronotum anteriorly narrowed, lateral edges slightly curved. Metanotum with slight postero-medial projection. Dorsal surface with dark brown maculation as on Fig. 4A and covered with fine, hair-like setae (as on abdominal terga and head); sparse longer, hair-like setae along pro-, meso- and metanotal suture.

***Legs*.** Colour pattern of femora as on Fig. 4F. Femora without medial hypodermal spot. Femora apically slightly darkened; patella-tibial suture darkened; tarsi proximally and distally darkened. Dorsal surface of femora covered by short (sporadically elongated) apically rounded spatulate setae (Fig. 5D). Dorsal margin of tibiae and tarsi with row of long setae; ventral margin of both with short distally accumulated spine-like setae. Tarsal claws with 3–4 denticles.

***Abdominal terga*.** Colour pattern of abdominal terga consists of transversal stripe along anterior margin of terga I–IX, medially extending to: i) large median triangular macula on terga II–III (IV), and ii) triangular or T-shaped macula on terga V–IX (medial macula on tergum VIII and IX often widened). Median macula on terga V–VII surrounded by pale area (Fig. 4G). Tergum X without distinct maculation. Lateral margin of terga I–IX with oblique macula. Denticles along posterior margin on terga strongly sclerotised, irregular and pointed (Fig. 5E). Surface of terga covered with hair-like setae and sparsely with stick-like setae. Tergum X with short postero-lateral projections (Fig. 5J, K). Longitudinal medial row of hair-like setae along abdominal terga present.

***Abdominal sterna*.** Yellowish, with colouration pattern on sterna I–VIII consisting of rounded median macula (Fig. 4H, arrow). In more pigmented specimens, median macula with paired pale spots located medio-posteriorly. Rounded median macula often poorly expressed and only medio-posterior edge of sterna is slightly pigmented (Fig. 4I, arrow). Colouration pattern sporadically restricted to sterna I and II or absent. Sternum IX of female with V-shaped median emargination and surface covered by setae centrally (Fig. 5L).

***Gills*.** Dorsal surface of gill plate I yellowish; of gill plates II–VII greyish on anterior half, brownish on posterior half. Ventral margin of all gill plates yellowish. Costal projection on gill plate III well-developed (Fig. 5G, arrow). Gill plate VII wide (in natural position of ventral view; Figs 4J, 5H, I).

***Cerci*.** Yellowish brown, basally darkened.

##### Subimagoes, imagoes, and eggs.

Unknown.

##### Morphological diagnostics of larvae.

Epeorus (Caucasiron) abditus sp. nov. can be distinguished by the combination of the following morphological characters: i) femora without median spot (Fig. 4F); ii) abdominal sterna with circular median macula as on Fig. 4H (colouration may be restricted to medio-posterior part of sterna as on Fig. 4I); iii) abdominal terga V–VII with triangular or T-shaped macula surrounded by pale area (Fig. 4G); iv) tergum X with short postero-lateral projection (Fig. 5J, K, arrow); v) surface of abdominal terga with hair-like setae (Fig. 5E); vi) shape of gill plates VII wide (in natural position from ventral view, Figs 4J; 5H, I).

##### Morphological affinities.

Epeorus (Caucasiron) abditus sp. nov. is similar to several species from the Caucasus and neighbouring Mediterranean and Irano-Anatolian ranges, namely E. (C.) alpestris (distributed in the Greater Caucasus), E. (C.) alborzicus Hrivniak & Sroka, 2020 (Alborz Mountains), and E. (C.) bicolliculatus Hrivniak, 2017 (Pontic Mountains, Lesser and Greater Caucasus). All of them possess abdominal sterna with a rounded median macula and femora without median spot (Hrivniak et al. 2020b).

Epeorus (C.) alpestris can be distinguished from E. (C.) abditus sp. nov. by the absence of postero-lateral projections on the tergum X (Hrivniak et al. 2020b, fig. 17L) present in E. (C.) abditus sp. nov. (Fig. 5J, K, arrow). Additionally, the rounded maculae on abdominal sterna are always present in E. (C.) alpestris (Hrivniak et al. 2020b, fig. 16I), whereas the colouration pattern of abdominal sterna varies from a well-defined pattern (Fig. 4H) to an indistinct (Fig. 4I) or no pattern in E. (C.) abditus sp. nov. Moreover, Epeorus (C.) alpestris is characterised by typical maculation of abdominal terga (Hrivniak et al. 2020b, fig. 16G, H).

Epeorus (C.) alborzicus possesses abdominal sterna with a large circular medial macula (Hrivniak et al. 2020b, fig. 40L–N) and blurred macula (or a pair of rounded maculae) on tergum II and III (Hrivniak et al. 2020b, fig. 40H, I), in contrast to E. (C.) abditus sp. nov. with relatively small rounded medial macula on abdominal sterna (Fig. 4H, I) and triangular macula on tergum II and III (Fig. 4G).

Epeorus (C.) bicolliculatus differs from E. (C.) abditus sp. nov. by the presence of paired postero-medial protuberances on abdominal terga II–IX (Hrivniak et al. 2020b, fig. 34H) and basally widened setae on the surface of terga (Hrivniak et al. 2020b, fig. 35E) in contrast to basally narrow setae in E. (C.) abditus sp. nov. (Fig. 5E).

The larvae of E. (C.) abditus sp. nov. with weakly pigmented abdominal sterna may be erroneously assigned to E. (C.) magnus (distributed in the Greater and Lesser Caucasus, Pontic and Taurus Mountains). This species differs from E. (C.) abditus sp. nov. by the presence of dense bristle-like setae on the dorsal surface of the labrum (Hrivniak et al. 2020b, fig. 11A) in contrast to sparse and hair-like setae in E. (C.) abditus sp. nov. (Fig. 5A). In addition, E. (C.) magnus often has clearly developed lateral projections on the tergum X (Hrivniak et al. 2020b, fig. 11K–M) in contrast to only short projections in E. (C.) abditus sp. nov. (Fig. 5J, K).

Two species distributed in the western and central Greater Caucasus, namely E. (C.) soldani (Braasch, 1979) and E. (C.) sinitshenkovae (Braasch & Zimmerman, 1979), have abdominal sterna without or with weakly developed colouration pattern and no femoral spot. Epeorus (C.) soldani can also be easily distinguished from E. (C.) abditus sp. nov. by setae on abdominal terga that are basally widened in the former species (Hrivniak et al. 2020b, fig. 20E) and narrow in the latter (Fig. 5E). Epeorus (C.) sinitshenkovae can be separated by a poorly developed projection on costal margin of gill plates (Hrivniak et al. 2020b, fig. 26G) from E. (C.) abditus sp. nov. bearing a well-developed projection (Fig. 5G). Additionally, E. (C.) sinitshenkovae is characterised by a specific colouration of abdominal terga and femora (Hrivniak et al. 2020b, fig. 25H, F).

All other species of E. (Caucasiron) from the Caucasus, Mediterranean, and Irano-Anatolian ranges can be easily distinguished from E. (C.) abditus sp. nov. by the presence of specific colouration pattern of abdominal sterna and/or presence of femoral spot. These include E. (C.) caucasicus (Tshernova, 1938), E. (C.) nigripilosus (Sinitshenkova, 1976), E. (C.) zagrosicus Hrivniak & Sroka, 2020, E. (C.) iranicus (Braasch & Soldán, 1979), E. (C.) longimaculatus (Braasch, 1980), E. (C.) turcicus Hrivniak, Türkmen & Kazancı, 2019, E. (C.) shargi, E. (C.) hyrcanicus, and E. (C.) tripertitus.

### ﻿Supplement to the identification guide to larvae of Caucasian and Irano-Anatolian species of E. (Caucasiron)

The identification guide by Hrivniak et al. (2020b) includes 15 species of E. (Caucasiron) described between 1938 and 2020 and covers the entire area of the Caucasus and adjacent Mediterranean and Irano-Anatolian mountain ranges. Since then, three more species have been described from this area, namely E. (C.) hyrcanicus, E. (C.) tripertitus, and E. (C.) abditus sp. nov. Therefore, we provide a supplement to the original guide that includes recently described species. For accurate identification of E. (Caucasiron) larvae, this supplement should be used prior to the original guide. When the possibility that the specimens to be identified represent one of the three recently described species is ruled out, the user can proceed with Hrivniak et al. (2020b). The abbreviations used in this key: N: north, SE: southeast, NE: northeast, SW: southwest. The geographic delimitation of the mountain ranges was given by Hrivniak et al. (2020b).

### ﻿Key to species (part II)

**Table:** 

1	Medial hypodermal femur spots present (Figs 6A, B, 7A)	**group A**
2	Medial hypodermal femur spots absent (Fig. 4F)	**group B**
Group A
–	Colouration pattern on abdominal sterna present (Figs 6D, E, 7D, E)	**3**
–	Colouration pattern on abdominal sterna absent	**continue to subgroup A2 in Hrivniak et al. (2020b, p. 9)**
3	Setae on abdominal terga wide at base (Fig. 6C). Sterna II–VI with rounded (or blurred) median macula and pair of medio-lateral maculae (Fig. 6D, arrows) (colouration pattern sometimes poorly expressed, Fig. 6E); gill plates VII narrow (Fig. 6F); medial hypodermal femur spot elongated, often blurred or poorly expressed (Fig. 6A, B)	**E. (C.) tripertitus (Greater Caucasus; see Hrivniak et al. 2022)**
4	Setae on abdominal terga hair-like (Fig. 7B). Sterna with oblique stripes often laterally extended (Fig. 7D, E, arrows); gill plates VII relatively wide (Fig. 7C); medial hypodermal femur spot as in Fig. 7A	**E. (C.) hyrcanicus (N Iran, SE Azerbaijan; see Hrivniak et al. 2021)**
–	Characters differ from the combinations above	**continue to subgroup A1 in Hrivniak (2020, p. 9)**
Group B
–	Sterna II–VI: with rounded median macula (Fig. 4H, arrow)/with darkened posterior margin (Fig. 4I, arrow)/unpatterned (sterna I–II often darkened); tergum X with short postero-lateral projections (Fig. 5J, K); gill plates VII wide (Figs 4J, 5H, I); dorsal margin of labrum with sparse setae (Fig. 5A)	**E. (C.) abditus sp. nov. (NE Türkiye, SW Georgia, Greater Caucasus)**
–	Characters differ from the combination above	**continue to group B in Hrivniak et al. (2020b, p. 9)**

**Figure 6.**
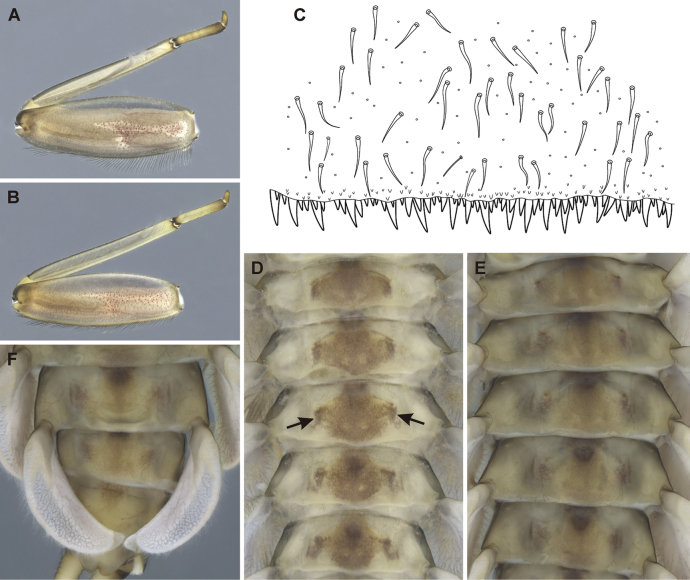
Epeorus (Caucasiron) tripertitus, larva **A, B** middle leg **C** surface and posterior margin of abdominal tergum VII **D, E** abdominal sterna II–VI (arrows point on paired medio-lateral maculae) **F** gills VII (in natural position from ventral view).

**Figure 7.**
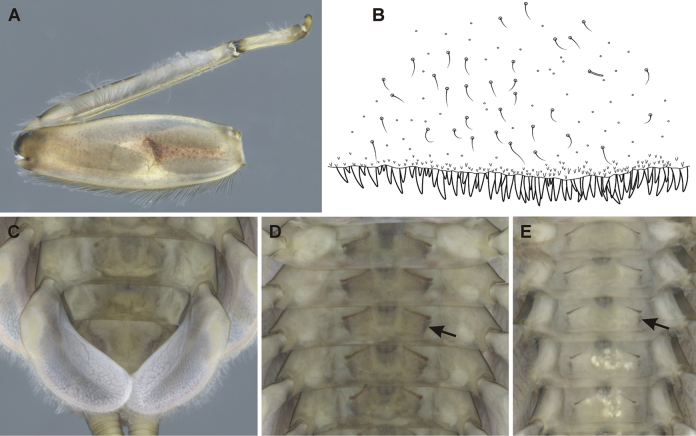
Epeorus (Caucasiron) hyrcanicus, larva **A** middle leg **B** surface and posterior margin of abdominal tergum VII **C** gills VII (in natural position from ventral view) **D, E** abdominal sterna II–VI (arrow points on lateral extension of oblique stripe).

## Supplementary Material

XML Treatment for Epeorus (Caucasiron) abditus

## ﻿Additional information

### Author ORCIDs

Ľuboš Hrivniak https://orcid.org/0000-0002-9327-1314

Pavel Sroka https://orcid.org/0000-0003-4367-6564

Gencer Türkmen https://orcid.org/0000-0002-1155-8275

Alexander V. Martynov https://orcid.org/0000-0002-6506-5134

### Data availability

All of the data that support the findings of this study are available in the main text.

## References

[B1] Bojková J Sroka P Soldán T Namin JI Staniczek AH Polášek M Hrivniak Ľ Abdoli A Godunko RJ (2018) Initial commented checklist of Iranian mayflies, with new area records and description of *Procloeoncaspicum* sp. n. (Insecta, Ephemeroptera, Baetidae). ZooKeys 749: 87–123. 10.3897/zookeys.749.24104 PMC5904425 29674922

[B2] Bouckaert R Heled J Kühnert D Vaughan T Wu CH Xie D Suchard MA Rambaut A Drummond AJ (2014) BEAST 2: a software platform for Bayesian evolutionary analysis. PLoS Computational Biology 10: e1003537. 10.1371/journal.pcbi.1003537 PMC3985171 24722319

[B3] Braasch D (1978) *Epeorusznojkoi* and *Ironmagnus* new species (HeptageniidaeEphemeroptera) from the Caucasus USSR. Entomologische Nachrichten 22(5): 65–70.

[B4] Braasch D (1979) Beitrag zur Kenntnis der Gattung Iron Eaton im Kaukasus (UdSSR) (III) (Ephemeroptera, Heptageniidae). Reichenbachia 17(33): 283–294.

[B5] Braasch D (1980) Beitrag zur Kenntnis der Gattung Iron Eaton (Heptageniidae, Ephemeroptera) im Kaukasus (UdSSR), 2. Entomologische Nachrichten 24(10–11): 166–173.

[B6] Braasch D (2006) Neue Eintagsfliegen der Gattungen *Epeorus* und *Iron* aus dem Himalaja (Ephemeroptera, Heptageniidae). Entomologische Nachrichten 50(1–2): 79–88.

[B7] Braasch D Zimmermann W (1979) Ironsinitshenkovae sp. n. – eine neue Heptageniide (Ephemeroptera) aus dem Kaukasus. Entomologische Nachrichten 23(7): 103–107.

[B8] Chen P Wang YY Zhou CF (2010) A new mayfly species of Epeorus (Caucasiron) from southwestern China (Ephemeroptera: Heptageniidae). Zootaxa 2527: 61–68. 10.11646/zootaxa.2527.1.4

[B9] Eaton AE (1881) An announcement of new genera of the Ephemeridae. Entomologist’s Monthly Magazine 18: 21–27.

[B10] Fujisawa T Barraclough TG (2013) Delimiting species using single-locus data and the generalized mixed yule coalescent approach: a revised method and evaluation on simulated datasets. Systematic Biology 62: 707–724. 10.1093/sysbio/syt033 23681854 PMC3739884

[B11] Hrivniak Ľ (2020) Diversity and speciation of mayflies (Ephemeroptera) from the Caucasus and adjacent areas. Ph.D. Thesis Series, No. 15. University of South Bohemia, Faculty of Science, School of Doctoral Studies in Biological Sciences, České Budějovice, 269 pp.

[B12] Hrivniak Ľ Sroka P Godunko RJ Žurovcová M (2017) Mayflies of the genus *Epeorus* Eaton, 1881 s.l. (Ephemeroptera: Heptageniidae) from the Caucasus Mountains: a new species of *Caucasiron* Kluge, 1997 from Georgia and Türkiye. Zootaxa 4341: 353–374. 10.11646/zootaxa.4341.3.2 29245660

[B13] Hrivniak Ľ Sroka P Godunko RJ Palatov D Polášek M Manko P Oboňa J (2018) Diversity of Armenian mayflies (Ephemeroptera) with the description of a new species of the genus *Ecdyonurus* (Heptageniidae). Zootaxa 4500: 195–221. 10.11646/zootaxa.4500.2.3 30486057

[B14] Hrivniak Ľ Sroka P Türkmen G Godunko RJ Kazancı N (2019) A new Epeorus (Caucasiron) (Ephemeroptera: Heptageniidae) species from Turkey based on molecular and morphological evidence. Zootaxa 4550: 58–70. 10.11646/zootaxa.4550.1.2 30790876

[B15] Hrivniak Ľ Sroka P Bojková J Godunko RJ Soldán T Staniczek AH (2020a) The impact of Miocene orogeny for the diversification of Caucasian Epeorus (Caucasiron) mayflies (Ephemeroptera: Heptageniidae). Molecular Phylogenetics and Evolution 146: 106735. 10.1016/j.ympev.2020.106735 32001364

[B16] Hrivniak Ľ Sroka P Bojková J Godunko RJ (2020b) Identification guide to larvae of Caucasian Epeorus (Caucasiron) (Ephemeroptera, Heptageniidae). ZooKeys 986: 1–53. 10.3897/zookeys.986.56276 33223879 PMC7661483

[B17] Hrivniak Ľ Sroka P Bojková J Godunko RJ Namin JI Bagheri S Nejat F Abdoli A Staniczek AH (2020c) Diversity and distribution of Epeorus (Caucasiron) (Ephemeroptera, Heptageniidae) in Iran, with descriptions of three new species. ZooKeys 947: 71–102. 10.3897/zookeys.947.51259 32733130 PMC7363713

[B18] Hrivniak Ľ Sroka P Bojková J Manko P Godunko RJ (2021) A new species of Epeorus (Caucasiron) (Ephemeroptera: Heptageniidae) from Azerbaijan and Iran. ZooKeys 1068: 13–26. 10.3897/zookeys.1068.70717 34790025 PMC8578148

[B19] Hrivniak Ľ Sroka P Godunko RJ Manko P Bojková J (2022) Diversification in Caucasian Epeorus (Caucasiron) mayflies (Ephemeroptera: Heptageniidae) follows topographic deformation along the Greater Caucasus range. Systematic Entomology 47(4): 603–617. 10.1111/syen.12551

[B20] Kapli T Lutteropp S Zhang J Kobert K Pavlidis P Stamatakis A Flouri T (2016) Multi-rate Poisson tree processes for single-locus species delimitation under maximum likelihood and Markov chain Monte Carlo. Bioinformatics 33(11): 1630–1638. 10.1093/bioinformatics/btx025 PMC5447239 28108445

[B21] Kluge NJ (1997) New subgenera of Holarctic mayflies (Ephemeroptera: Heptageniidae, Leptophlebiidae, Ephemerellidae). Zoosystematica Rossica 5(2): 233–235.

[B22] Kluge NJ (2015) Central Asian mountain Rhithrogenini (Ephemeroptera: Heptageniidae) with pointed and ephemeropteroid claws in the winged stages. Zootaxa 3994: 301–353. https://biotaxa.org/Zootaxa/article/view/zootaxa.3994.3.1 26250277 10.11646/zootaxa.3994.3.1

[B23] Kumar S Stecher G Li M Knyaz C Tamura K (2018) MEGA X: Molecular Evolutionary Genetics Analysis across computing platforms. Molecular Biology and Evolution 35: 1547–1549. 10.1093/molbev/msy096 29722887 PMC5967553

[B24] Ma Z Li R Zhu B Zheng X Zhou C (2022) Comparative mitogenome analyses of subgenera and species groups in *Epeorus* (Ephemeroptera: Heptageniidae). Insects 13: 599. 10.3390/insects13070599 PMC9317806 35886775

[B25] Miller MA Pfeiffer W Schwartz T (2010) Creating the CIPRES Science Gateway for inference of large phylogenetic trees. In: Proceedings of the Gateway Computing Environments Workshop (GCE), 14 Nov. 2010, New Orleans, 1–8. 10.1109/GCE.2010.5676129

[B26] Mittermeier RA Turner WR Larsen FW Brooks TM Gascon C (2011) Global biodiversity conservation: the critical role of hotspots. In: Zachos FE, Habel JC (Eds) Biodiversity Hotspots. Distribution and Protection of Conservation Priority Areas. Springer, Berlin, Heidelberg.

[B27] Pons J Barraclough TG Gomez-Zurita J Cardoso A Duran DP Hazell S Kamoun S Sumlin WD Vogler AP (2006) Sequence-based species delimitation for the DNA taxonomy of undescribed insects. Systematic Biology 55: 595–609. 10.1080/10635150600852011 16967577

[B28] Puillandre N Brouillet S Achaz G (2021) ASAP: Assemble species by automatic partitioning. Molecular Ecology Resources 21: 609–620. 10.1111/1755-0998.13281 33058550

[B29] Rambaut A Drummond AJ Xie D Baele G Suchard MA (2018) Posterior summarisation in Bayesian phylogenetics using Tracer 1.7. Systematic Biology 67(5): 901–904. 10.1093/sysbio/syy032 29718447 PMC6101584

[B30] Sinitshenkova ND (1976) Mayflies of the genus Iron Eaton (Ephemeroptera, Heptageniidae) in the fauna of the Caucasus. Entomological Review 55(4): 85–92.

[B31] Tshernova OA (1938) Zur Kenntnis der Ephemeropteren Ost-Transkaukasien. Trudy Azerbajdshanskogo Filiala AN SSSR, Baku 7(42): 55–64.

[B32] Tshernova OA (1981) On the systematics of adult mayflies of the genus *Epeorus* Eaton 1881 (Ephemeroptera, Heptageniidae). Revue d’Entomologie de l’URSS] 60(2): 323–336.

[B33] Waterhouse AM Procter JB Martin DMA Clamp M Barton GJ (2009) Jalview Version 2 – a multiple sequence alignment editor and analysis workbench. Bioinformatics 25: 1189–1191. 10.1093/bioinformatics/btp033 19151095 PMC2672624

